# Boosting the Performance of Photomultiplication‐Type Organic Photodiodes by Embedding CsPbBr_3_ Perovskite Nanocrystals

**DOI:** 10.1002/advs.202305349

**Published:** 2023-12-08

**Authors:** Mingyun Kang, Dong Hyeon Lee, Juhee Kim, Geon‐Hee Nam, Seyeon Baek, Seongmin Heo, Yong‐Young Noh, Dae Sung Chung

**Affiliations:** ^1^ Department of Chemical Engineering Pohang University of Science and Technology (POSTECH) Pohang 37673 Republic of Korea

**Keywords:** electrostatic interactions, nanocrystals, organic photodiodes, perovskites, photomultiplication

## Abstract

In this study, it is demonstrated that CsPbBr_3_ perovskite nanocrystals (NCs) can enhance the overall performances of photomultiplication‐type organic photodiodes (PM‐OPDs). The proposed approach enables the ionic‐polarizable CsPbBr_3_ NCs to be evenly distributed throughout the depletion region of Schottky junction interface, allowing the entire trapped electrons within the depletion region to be stabilized, in contrast to previously reported interface‐limited strategies. The optimized CsPbBr_3_‐NC‐embedded poly(3‐hexylthiophene‐diyl)‐based PM‐OPDs exhibit exceptionally high external quantum efficiency, specific detectivity, and gain–bandwidth product of 2,840,000%, 3.97 × 10^15^ Jones, and 2.14 × 10^7^ Hz, respectively. 2D grazing‐incidence X–ray diffraction analyses and drift–diffusion simulations combined with temperature‐dependent *J–V* characteristic analyses are conducted to investigate the physics behind the success of CsPbBr_3_‐NC‐embedded PM‐OPDs. The results show that the electrostatic interactions generated by the ionic polarization of NCs effectively stabilize the trapped electrons throughout the entire volume of the photoactive layer, thereby successfully increasing the effective energy depth of the trap states and allowing efficient PM mechanisms. This study demonstrates how a hybrid‐photoactive‐layer approach can further enhance PM‐OPD when the functionality of inorganic inclusions meets the requirements of the target device.

## Graphical abstract

A hybrid approach of embedding perovskite nanocrystals (NCs) into photoactive layers of photomultiplication‐type organic photodiodes (PM‐OPDs) is proposed. Embedded perovskite NCs polarize via migration of their constituting ions in the presence of an external applied bias and supply electrostatic interactions to trapped electrons over the entire volume of the photoactive layer, thereby resulting in ultrahigh performances of the optimized PM‐OPD.

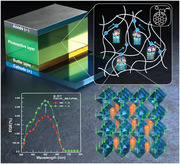

## 1 Introduction

Organic photodiodes (OPDs), in which organic semiconductors are employed as photoactive materials, have been actively studied as potential alternatives to conventional Si‐based photodiodes. Organic semiconductors offer the following advantages: 1) color‐selective absorption, 2) thin photoactive layers originating from high absorption coefficients, and 3) the possibility of flexible (e.g., foldable and rollable) applications.^[^ ^1^, ^2^, ^3^, ^4^, ^5^, ^6^, ^7^, ^8^, ^9^, ^10^, ^11^, ^12^, ^13^, ^14^ ^]^ In particular, photomultiplication‐type OPDs (PM‐OPDs) have attracted considerable attention, not only in conventional camera industries but also in industries such as healthcare (pulse oximeters), security (biometric sensors for iris, vein, or fingerprint recognition), and optical communication because their self‐amplifying characteristics enables them to detect extremely weak light sources. To date, most PM‐OPDs reported in the literature exhibit high external quantum efficiencies (EQEs) of more than 10000%, which are related to photoconductive gain.^[^ ^5^, ^6^, ^15^, ^16^, ^17^, ^18^, ^19^, ^20^, ^21^, ^22^, ^23^ ^]^

The term “gain” is defined as an index that describes the number of conducting charges generated by a single incident photon. Recent reports regarding the generation of intrinsic and extrinsic gains show that the gain of a photoconductor with a majority of hole carriers can be expressed as follows:

(1) $$G\;=\frac{\tau_{r}}{\tau_{\mathrm{transit}}}\;\left(1+\frac{\Delta p}{\Delta n}\frac{\mu_{p}}{\mu_{n}}\right)$$

where *τ*_r_ is the minority carrier recombination lifetime, *τ*_transit_ is the carrier transit time, Δ*p* and Δ*n* are the excess hole and electron densities, respectively, and *µ*_p_ and *µ*_n_ are the hole and electron mobilities, respectively.^[^ ^5^, ^6^, ^24^ ^]^ This implies that intentionally created energetically and spatially efficient minority carrier (in this case, electrons) traps can lead to a longer carrier recombination lifetime (intrinsic gain), higher excess hole‐to‐electron concentration ratio (extrinsic gain), and higher hole‐to‐electron mobility ratio (extrinsic gain), which results in a high device gain and EQE. In conventional PM‐OPDs with holes as the majority carriers,^[^ ^15^, ^17^, ^18^, ^19^, ^20^, ^21^, ^22^, ^23^ ^]^ the photoactive layers primarily consist of an electron donor polymer, poly(3‐hexylthiophene‐diyl) (P3HT), which controls photon absorption, exciton formation, and charge carrier transportation, with a small amount of an electron acceptor molecule, [6,6]‐phenyl‐C_71_‐butyric acid methyl ester (PC_71_BM). Semiconductor–Al Schottky junctions are typically employed in the conventional device architecture of indium tin oxide (ITO)/hole transport layer/photoactive layer/Al. However, the effective electron traps are located at the far end of the optical propagation pathway (semiconductor–Al interface); therefore, this structure is limited because the spectral shapes of the PM‐OPD photoresponse are reversed compared to the absorption spectrum of the matrix donor polymer, and the EQE is restricted to ≈100000%.^[^ ^15^, ^17^, ^18^, ^19^, ^20^, ^21^, ^22^, ^23^ ^]^

To address the challenges encountered by conventional PM‐OPDs, we recently used the concept of “electrostatic interaction” by introducing a polyelectrolyte as an electric double layer (EDL) on top of the ITO cathode layer,^[^ ^6^ ^]^ which enabled the formation of an ITO–semiconductor Schottky junction at an optically favorable position and induced electrostatic interactions between the exposed cations of the EDL and trapped electrons in the photoactive acceptor material domains at the EDL–photoactive layer interface. The resulting PM‐OPD exhibited green‐selective photoresponses, which resembled the absorption spectrum of the photoactive donor P3HT, and exceptionally high EQEs with an average value of ≈2000000% was achieved. Although these interfacial electrostatic interactions are effective in realizing high EQE PM‐OPDs, this approach limits the electrostatic interactions to the vicinity of the EDL. Therefore, it is essential to generate electrostatic interactions within the entire volume of the photoactive layer to further enhance PM‐OPD performances.

Herein, we introduce CsPbBr_3_ perovskite nanocrystals (NCs) as additives to the organic photoactive layers of PM‐OPDs. Several studies have been conducted using perovskite NCs as additives in organic semiconductors to significantly improve photoelectric performances.^[^ ^25^, ^26^, ^27^ ^]^ However, unlike those, the proposed approach takes advantage of ion migration in perovskites, which is typically considered a critical drawback. Light exposure is a major cause of ion migration. Previous reports have shown that ion migration occurs readily under illumination,^[^ ^28^, ^29^, ^30^, ^31^ ^]^ and even more frequently in mixed halide perovskites.^[^ ^32^, ^33^ ^]^ Another major cause of ion migration is an externally applied bias, which is known to induce the structural degradation of perovskites, thereby reducing device performances.^[^ ^28^, ^29^, ^34^, ^35^, ^36^, ^37^ ^]^ This degradation issue can be observed in both bulk perovskite phases and NCs.^[^ ^28^, ^29^, ^38^, ^39^, ^40^, ^41^ ^]^ In the present study, the ion migration occuring in the presence of a high external bias was utilized to initiate additional electrostatic interactions to trapped electrons in photoactive layers, which is the key to photoconductive gain generation.

**Figure 1:**
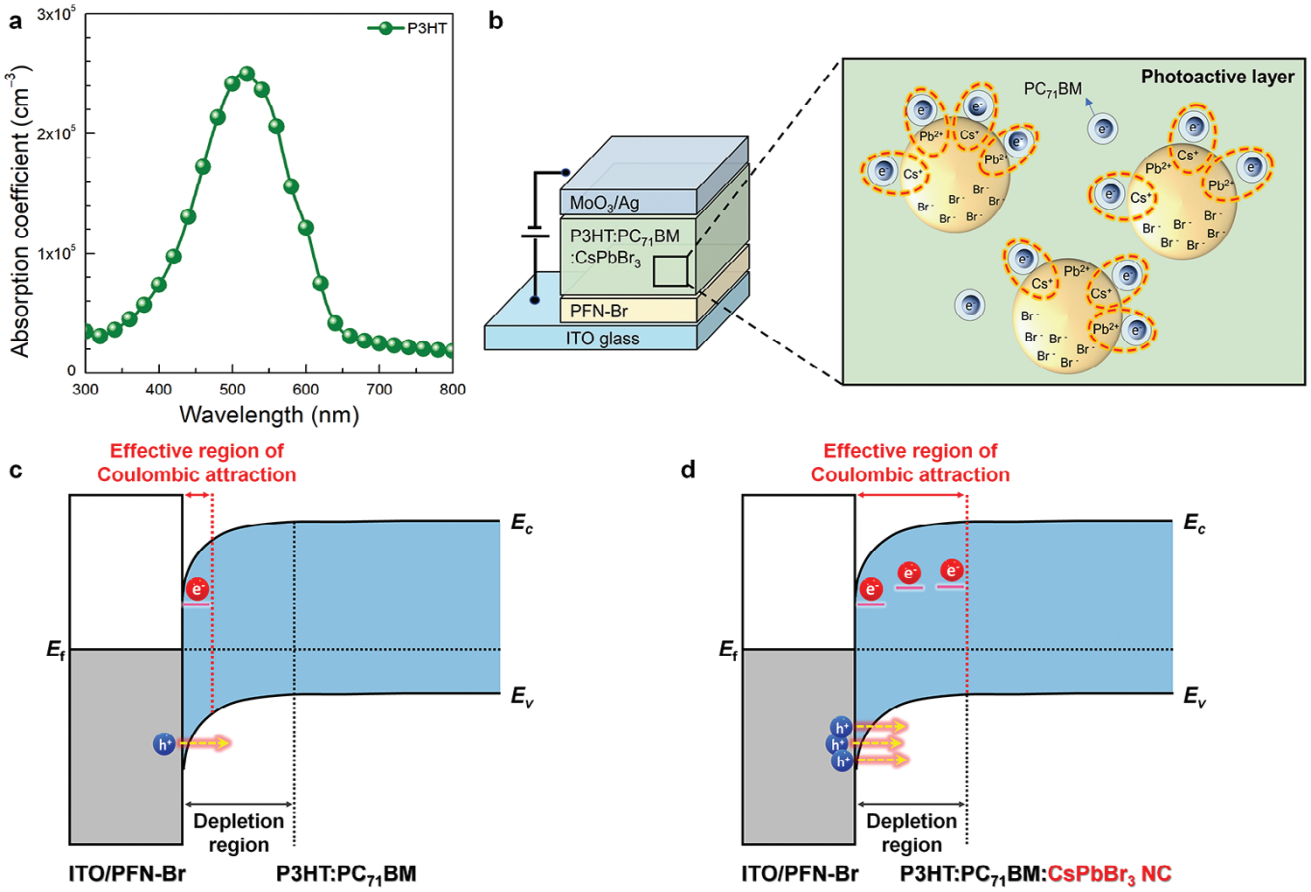
a) Absorption spectrum of P3HT. b) device structure of the PM‐OPD based on the P3HT:PC_71_BM:CsPbBr_3_ NC ternary blend, in which positively polarized CsPbBr_3_ NCs within the photoactive layer trigger electrostatic interactions to electrons trapped in the PC_71_BM domains. c,d) Energy band diagram of c) the electric‐double‐layer‐based and d) ionic‐polarizable‐nanocrystal‐embedded PM‐OPDs under illumination.

As schematically illustrated in **Figure** **1**, when CsPbBr_3_ perovskite NCs are incorporated in the photoactive layer of ITO/poly(9,9‐bis(3′‐(*N*,*N*‐dimethyl)‐*N*‐ethylammonium‐propyl‐2,7‐fluorene)‐*alt*‐2,7‐(9,9‐dioctylfluorene))dibromide (PFN–Br)/P3HT:PC_71_BM (100:1, w/w)/MoO_3_/Ag, electrostatic interactions can be expected between positively polarized CsPbBr_3_ perovskite NCs and electrons trapped in the PC_71_BM domains, which is described in detail using an energy diagram in Figure S1 (Supporting Information). In previous EDL‐based PM‐OPDs,^[^ ^6^ ^]^ where only interfacial electrostatic interactions existed, the depletion region is obviously wider than the effective region of Coulombic attraction driven by ionic interlayer (Figure 1c); however, the proposed system in the present study enables electrostatic interactions within the entire volume of the photoactive layer, which strengthens its electron‐trapping ability (Figure 1d). Then, all the trapped electrons in the entire depletion region will cause Coulombic attraction, enhancing the hole injection from the ITO/PFN–Br cathode. 2D grazing‐incidence X–ray diffraction (2D‐GIXD) experiments were conducted to analyze the morphological effects of incorporating perovskite NCs into the photoactive layer. Drift–diffusion simulations combined with temperature‐dependent *J–V* characteristic analyses were also conducted to show that the electrostatic interactions within the photoactive layer effectively stabilized the electron trapping of PC_71_BM domains and increased the effective energy trap depth. The optimized CsPbBr_3_‐NC‐embedded PM‐OPD achieved a significantly high EQE, specific detectivity, and gain–bandwidth (*G–B*) product of 2840000%, 3.97×10^15^ Jones, and 2.14×10^7^ Hz, respectively. Note that the specific detectivity and *G–B* product values realized in the present work are currently the highest among those reported for visible organic/hybrid photodetectors.

## 2 Results and Discussion

### 2.1 Morphological Optimization

**Figure 2:**
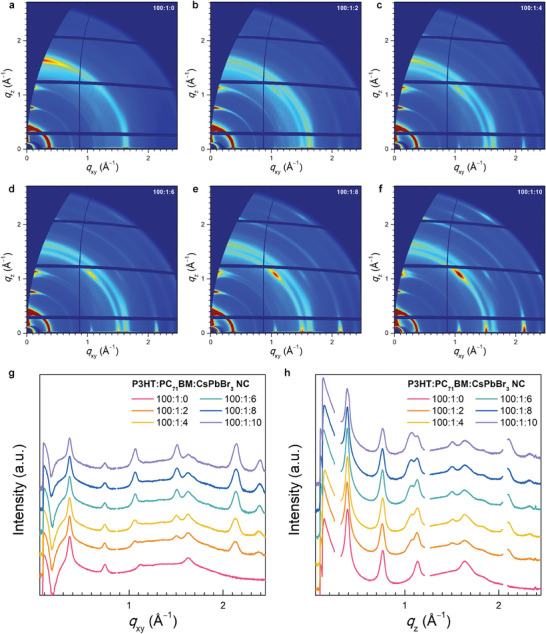
a–f) 2D‐GIXD patterns of P3HT:PC_71_BM:CsPbBr_3_ NC (100:1:X, w/w; X = 0, 2, 4, 6, 8, and 10) blend films. g,h) Line‐cut profiles of P3HT:PC_71_BM:CsPbBr_3_ NC blend films along the g) *q*_xy_ and h) *q*_z_ axes.

The crystallinity of the photoactive blend thin film is governed by the concentration of CsPbBr_3_ NCs in the P3HT:PC_71_BM (100:1, w/w) ternary blend precursor. Introducing a large number of CsPbBr_3_ NCs is expected to induce a high degree of electrostatic interactions within the photoactive layer, thereby enhancing the photoconductive gain generation. However, the excess CsPbBr_3_ NC content may adversely affect the crystallinity of the P3HT:PC_71_BM thin film, resulting in the reduction of the generated photoconductive gain; cohesive stackings (both lamellar and *π*–*π*) of P3HT are disrupted because of its side chain interactions with aliphatic ligand chains attached on CsPbBr_3_ NC surfaces. Therefore, empirically determining the optimal CsPbBr_3_ NC content that can preserve the crystallinity of photoactive blend thin films is necessary. Thus, 2D‐GIXD measurements were performed on P3HT:PC_71_BM:CsPbBr_3_ NC (100:1:X, w/w; X = 0, 2, 4, 6, 8, and 10) blend films using the PLS‐II 3C beamline at the Pohang Accelerator Laboratory (PAL) in the Republic of Korea. **Figure** **2**a–f and S1 (Supporting Information) show the comparison of the 2D‐GIXD patterns of the P3HT:PC_71_BM:CsPbBr_3_ NC blend films. The P3HT:PC_71_BM (100:1, w/w) blend film clearly exhibited *π*–*π* stacking (010) and lamellar stacking (100)–(300) peaks in both the in‐plane and out‐of‐plane directions (Figure 2a–f). As the CsPbBr_3_ NC concentration increased, the crystalline feature of the CsPbBr_3_ NC film developed in the pattern of the ternary blend films, as shown in Figure S2 (Supporting Information), which is consistent with the corresponding line‐cut profiles along the *q*_xy_ and *q*_z_ axes presented in Figure 2g,h. Based on the positions of the *π*–*π* stacking (010) and lamellar stacking (100) peaks, the interlamellar spacing (*d*–spacing) and *π*–*π* stacking distance values were obtained as ≈16 and 3.84 Å, respectively, for all the P3HT:PC_71_BM:CsPbBr_3_ NC blend films. Because *π*–*π* stacking is intimately related to the charge transport in conjugated organic semiconductor films, the paracrystalline disorder parameter *g*_(010)_ for *π*–*π* stacking was calculated based on the single peak width estimation method, using the following equation:

(2) $$\;g_{\left(010\right)}=\sqrt{\frac{\Delta q}{2\pi q_{0}}}$$

where *Δq* is the width of the *π*–*π* stacking diffraction peak, and *q*_0_ is the center position of the *π*–*π* stacking peak.^[^ ^42^, ^43^ ^]^ The calculated *g*_(010),xy_/*g*_(010),z_ values were 5.50%/5.80%, 5.53%/5.85%, 5.56%/5.84%, 5.47%/5.86%, 5.76%/6.07%, and 6.49%/6.11% for the P3HT:PC_71_BM:CsPbBr_3_ NC (100:1:X, w/w; X = 0, 2, 4, 6, 8, and 10) blend films, respectively. All the morphological parameters of the ternary blend films are summarized in **Table** **1**. The table shows that the crystalline features of the ternary blend films deteriorated rapidly for the P3HT:PC_71_BM:CsPbBr_3_ NC blend composition of 100:1:8 and above. Therefore, from a structural perspective, the optimal P3HT:PC_71_BM:CsPbBr_3_ NC blend composition for the highest PM‐OPD performances was in the range of 100:1:2–100:1:6.

**Table 1:** Summary of packing parameters for P3HT:PC_71_BM:CsPbBr_3_ NC (100:1:X, w/w; X = 0, 2, 4, 6, 8, and 10) blend films, derived from 2D‐GIXD.

P3HT:PC_71_BM:CsPbBr_3_ NC	*d*–spacing [Å]	*π*–*π* stacking [Å]	*g*_(010),xy_ [%]	*g*_(010),z_ [%]
100:1:0	16.1	3.83	5.50	5.80
100:1:2	16.2	3.83	5.53	5.85
100:1:4	16.0	3.84	5.56	5.84
100:1:6	16.1	3.84	5.47	5.86
100:1:8	16.1	3.84	5.76	6.07
100:1:10	16.2	3.84	6.49	6.11

### 2.2 PM‐OPD Performances

**Figure 3:**
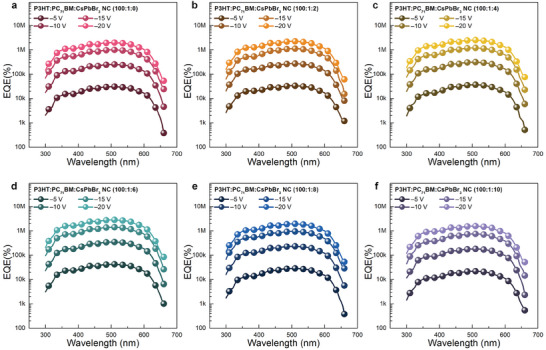
EQE spectra of the optimized PM‐OPDs based on the P3HT:PC_71_BM:CsPbBr_3_ NC (100:1:X, w/w; X = 0, 2, 4, 6, 8, and 10) ternary blend films, measured under different reverse biases (−5, −10, −15, and −20 V).

PM‐OPDs based on the P3HT:PC_71_BM:CsPbBr_3_ NC photoactive layer were fabricated with the device architecture ITO/PFN–Br/ P3HT:PC_71_BM:CsPbBr_3_ NC (100:1:X, w/w; X = 0, 2, 4, 6, 8, 10)/MoO_3_/Ag to evaluate the effect of CsPbBr_3_ NC concentration on the device performance. Dark and illuminated *J–V* characteristics of the PM‐OPDs are displayed in Figure S3 (Supporting Information). Dark current density values of the PM‐OPDs measured under a reverse bias of 5 V were 5.09 × 10^−6^, 4.12 × 10^−6^, 4.77 × 10^−6^, 3.48 × 10^−6^, 4.45 × 10^−6^, and 3.80 × 10^−6^ A cm^−2^ for P3HT:PC_71_BM:CsPbBr_3_ NC concentration ratios of 100:1:0, 100:1:2, 100:1:4, 100:1:6, 100:1:8, and 100:1:10, respectively, which did not differ significantly and had an average value of 4.29 × 10^−6^ A cm^−2^. The Schottky junction formed in the PM‐OPDs involved only the cathode (ITO/PFN–Br) and photoactive matrix (P3HT); therefore, negligible change in the dark current density was observed with the increasing CsPbBr_3_ NC content. However, presence of CsPbBr_3_ NCs within the photoactive layer triggered electrostatic interactions to the electrons trapped in the PC_71_BM domains owing to the polarization of CsPbBr_3_ NCs, which led to the stabilization of the effective electron traps that directly contributed to both intrinsic and extrinsic gain generation.^[^ ^6^, ^7^, ^44^, ^45^ ^]^ This phenomenon was observed in the illuminated *J–V* characteristics of the CsPbBr_3_‐NC‐free (100:1:0, w/w) and CsPbBr_3_‐NC‐embedded PM‐OPDs (100:1:X, w/w; X = 2, 4, 6, 8, 10). The extracted illuminated current density values were 1.61 × 10^−3^, 1.76 × 10^−3^, 1.94 × 10^−3^, 2.23 × 10^−3^, 1.51 × 10^−3^, and 1.15 × 10^−3^ A cm^−2^ for P3HT:PC_71_BM:CsPbBr_3_ NC concentration ratios of 100:1:0, 100:1:2, 100:1:4, 100:1:6, 100:1:8, and 100:1:10, respectively, under a 520‐nm illumination with a power density of 1.24 × 10^−5^ W cm^−2^. Contrary to the dark‐current results, the illuminated current density changed with the increasing CsPbBr_3_ NC concentration. The trend was in good agreement with that observed from the 2D‐GIXD analyses, thereby implying that the decreased crystallinity critically affected the optoelectronic performances, presumably by increasing *τ*_transit_ and decreasing *µ*_p_/*µ*_n_ in **Equation (1)**. This tendency was also reflected in the EQE spectra of the CsPbBr_3_‐NC‐embedded PM‐OPDs, as shown in **Figure** **3**. As summarized in **Table** **2**, the peak EQE values, measured under a reverse bias of −20 V, were 2000000%, 2250000%, 2540000%, 2840000%, 1960000%, and 1590000% for P3HT:PC_71_BM:CsPbBr_3_ NC concentration ratios of 100:1:0, 100:1:2, 100:1:4, 100:1:6, 100:1:8, and 100:1:10, respectively. Therefore, the optimal P3HT:PC_71_BM:CsPbBr_3_ NC concentration ratio for performance improvement was determined to be 100:1:6.

**Table 2:** Summary of peak EQE values obtained from the optimized PM‐OPDs based on P3HT:PC_71_BM:CsPbBr_3_ NC (100:1:X, w/w; X = 0, 2, 4, 6, 8, and 10) blend films.

P3HT:PC_71_BM:CsPbBr_3_ NC	@ −5 V [%]	@ −10 V [%]	@ −15 V [%]	@ −20 V [%]
100:1:0	30,800%	253,000%	1,010,000%	2,000,000%
100:1:2	33,700%	274,000%	1,130,000%	2,250,000%
100:1:4	37,100%	315,000%	1,210,000%	2,540,000%
100:1:6	42,500%	351,000%	1,420,000%	2,840,000%
100:1:8	28,800%	234,000%	953,000%	1,960,000%
100:1:10	22,000%	182,000%	758,000%	1,590,000%

### 2.3 Electrostatic Interaction Effect

It is known from previous studies that in the case of bulk perovskite materials, surface charges are generated by polarization upon external voltage application.^[^ ^46^ ^]^ In this study, the current density relaxation of the device at a short circuit condition after the accumulation of surface charges was observed. We have also conducted time‐resolved current density analyses with a CsPbBr_3_‐NC‐based hole‐only device using the device architecture of ITO/PEDOT:PSS/CsPbBr_3_ NC/MoO_3_/Ag. As shown in Figure S4 (Supporting Information), the measured interface charge density started to increase rapidly at 2 V and saturated at 4 V, with an average value of ≈84 µC cm^−2^, implying that CsPbBr_3_ NCs exhibit surface charges under an external applied bias.

**Figure 4:**
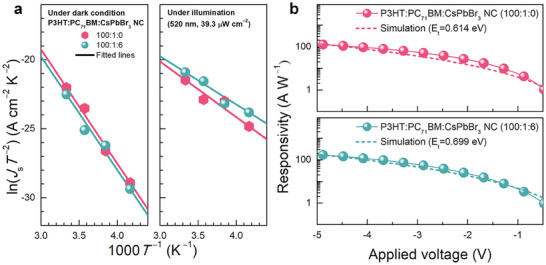
a) ln(*J*_s_ *T* ^−2^) versus *T* ^−1^ plots derived from the temperature‐dependent *J–V* curves of the PM‐OPDs with (100:1:6, w/w) and without (100:1:0, w/w) CsPbBr_3_ NC. b) Experimental (solid lines with symbols) and simulated (dotted lines) *R*( = *J*_ph_/*P*)*–V* curves of the CsPbBr_3_‐NC‐free and CsPbBr_3_‐NC‐free PM‐OPDs.

Then, we elucidated the electrostatic interaction effects of the strategically incorporated CsPbBr_3_ NCs on the stabilization of effective electron traps. The drift–diffusion simulations were conducted using SETFOS 5.2 (Fluxim AG, https://www.fluxim.com/setfos‐intro) to empirically fit the obtained data with the simulated data and determine the relevant fitting parameters (in this case, effective trap depths).^[^ ^47^ ^]^ Details related to the drift–diffusion simulation setup and procedures are described in the Supporting Information and Table S1 (Supporting Information). For more precise simulations, the effective Schottky junction barriers of the PM‐OPDs under both dark and illuminated conditions must be determined. We assumed that the carrier injection mechanism adheres to the thermionic emission model, which is typically utilized for Schottky junction barrier injection. The saturation current density (*J*_s_) is expressed as follows:

(3) $$\;J_{s}=A^{*}\;T^{2}\exp\left(\frac{-\varphi_{B}}{kT}\right)$$

where A* is the Richardson constant, *T* is the temperature, *φ*_B_ is the effective barrier height, and *k* is the Boltzmann constant.^[^ ^6^, ^15^, ^19^, ^48^ ^]^ Furthermore, *φ*_B_ can be obtained from the slope of the plot of ln(*J*_s_T−2) versus T−1. *J*_s_ can be determined from the ideal diode equation, as follows:

(4) $$J\;=J_{s}\;\left[\exp\left(\frac{qV}{kT}\right)-1\right]$$

where *J* is the current density, *q* is the elementary charge, and *V* is the applied voltage.^[^ ^6^, ^15^, ^19^, ^48^ ^]^ This equation can be further simplified for a sufficiently high applied positive bias as follows:

(5) $$J\;=J_{s}\;\exp\left(\frac{qV}{kT}\right)$$

where *J*_s_ is the *y*‐intercept of the line extrapolated from the high‐bias region of the *J–V* curve. Figure S5 (Supporting Information) shows the temperature‐dependent *J–V* curves of the optimized CsPbBr_3_‐NC‐embedded (100:1:6, w/w) and CsPbBr_3_‐NC‐free (100:1:0, w/w) PM‐OPDs under dark and illuminated conditions. The corresponding ln(*J*_s_ *T* ^−2^) versus *T* ^−1^ plots are shown in **Figure** **4**a. The *φ*_B_ values under dark/illuminated conditions for the CsPbBr_3_‐NC‐free and for the CsPbBr_3_‐NC‐embedded PM‐OPD were 0.713/0.348 eV and 0.709/0.301 eV, respectively. These results show that the polarized CsPbBr_3_ NCs promoted the PM‐OPD “Schottky junction barrier thinning” mechanism of the PM‐OPDs.^[^ ^5^, ^6^, ^7^, ^19^, ^49^ ^]^ Drift–diffusion simulations were then performed by determining the effective Fermi levels of the cathodes under dark and illuminated conditions based on the extracted *φ*_B_ values. Simulated *R–V* curves are shown in Figure 4b, which were used to fit the experimentally obtained *R–V* curves. The effective trap energy depth values with respect to the lowest unoccupied molecular orbital level of P3HT were 0.614 and 0.699 eV for the CsPbBr_3_‐NC‐free and CsPbBr_3_‐NC‐embedded PM‐OPDs, respectively, implying that electrostatic interactions strategically introduced within the photoactive layer efficiently stabilized the trapped electron states, which considerably improved the photoconductive gain generation.

### 2.4 Other Performances

**Figure 5:**
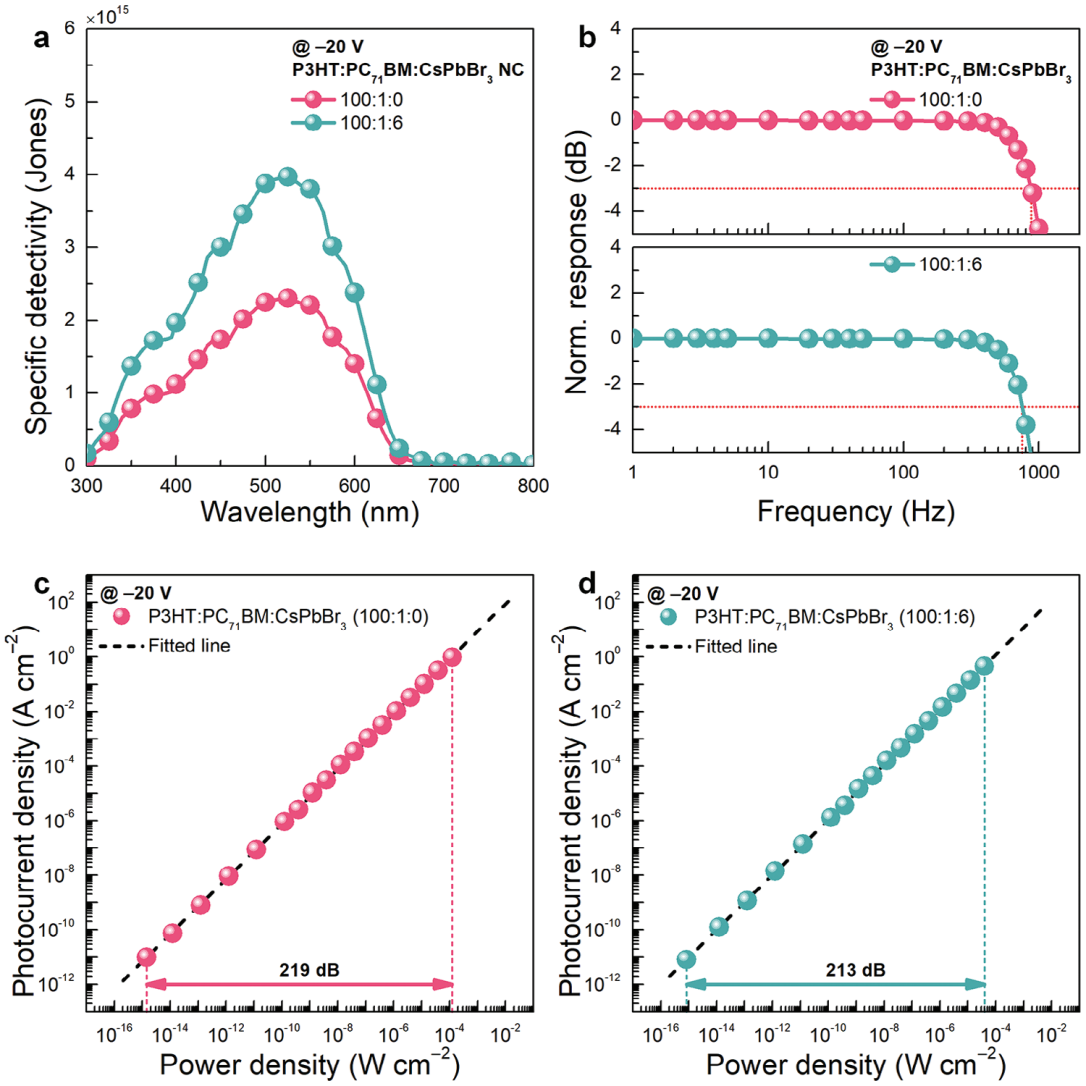
a) Specific detectivity spectra of the optimized PM‐OPDs with (100:1:6, w/w) and without (100:1:0, w/w) CsPbBr_3_ NC. b–d) Bode b) and *J*_ph_ *–P* c,d) plots of the optimized CsPbBr_3_‐NC‐free and CsPbBr_3_‐NC‐embedded PM‐OPDs. All measurements were conducted under a constant bias of −20 V.

Other photodiode performances including specific detectivity, linear dynamic range (LDR), and bandwidth were addressed to comprehensively evaluate the effects of internal electrostatic interactions on the optoelectronic properties of PM‐OPDs. Specific detectivity can be defined as follows:

(6) $$\;D^{*}=\frac{q\lambda \sqrt{A}\mathrm{EQE}}{hci_{\mathrm{noise}}}$$

where *λ* is the wavelength of the incident light, *A* is the photoactive area, *h* is Planck's constant, *c* is the speed of light, and *i*_noise_ is the noise current.^[^ ^2^, ^3^, ^4^, ^50^ ^]^ Based on the EQE spectra (Figure 2) and noise‐equivalent power (NEP) values (1.30 × 10^−16^ and 7.56 × 10^−17^ W Hz^−0.5^ for the CsPbBr_3_‐NC‐free and CsPbBr_3_‐NC‐embedded PM‐OPDs, respectively) extracted from the current spectral density plots (Figure S6, Supporting Information), the D* values at all wavelengths ranging from 300 to 800 nm were calculated, as shown in **Figure** **5**a. The calculated peak D* values for the optimized PM‐OPDs were 2.30 × 10^15^ and 3.97 × 10^15^ Jones for the CsPbBr_3_‐NC‐free and CsPbBr_3_‐NC‐embedded, respectively. Among D* values of the reported visible organic/hybrid photodetectors (≈10^14^ Jones),^[^ ^51^, ^52^, ^53^, ^54^ ^]^ which are not overestimated with the shot noise current calculation but empirically determined with the real noise current, the value for the CsPbBr_3_‐NC‐embedded PM‐OPD is currently the largest, owing to its enhanced internal electrostatic interactions. **Table** **3** summarizes the performances of previously reported PM‐OPDs that utilizes P3HT:PCBM as photoactive material. To confirm the universality of the ionic‐polarizable CsPbBr_3_ NC incorporation, PM‐OPDs were also fabricated with poly(9,9‐dioctylfluorene‐alt‐bithiophene) (F8T2) instead of P3HT. As shown in Figure S7 (Supporting Information), EQE value of the F8T2‐based PM‐OPD improved from 10300% to 23000% after the incorporation of ionic‐polarizable CsPbBr_3_ NCs. Operational stability test was conducted with the CsPbBr_3_‐NC‐embedded PM‐OPD by exposing it to a continuous light pulse of 1 Hz (520 nm, 4.89 × 10^−7^ W cm^−2^) for 25 h. Changes in the dark current and photocurrent densities were observed as displayed in Figure S8 (Supporting Information).

**Table 3:** Summary of performances for P3HT:PCBM‐based PM‐OPDs.

Structure	Applied bias [V]	EQE [%]	*D*_noise_* [Jones]	Reference
ITO/PFN–Br/ P3HT:PC_71_BM:CsPbBr_3_ NC/ MoO_3_/Ag	−20	2,840,000	3.97 × 10^15^ ^a^ ^)^	This work
ITO/PFN–Br/P3HT:PC_71_BM/ MoO_3_/Ag	−20	2,000,000	2.30 × 10^15^ ^a^ ^)^	This work
ITO/PEIE/ P3HT:PC_71_BM/ PEDOT:PSS/Ag	−20	12,000	1.48 × 10^14^ ^b)^	[55]
ITO/PFN–OX/ P3HT:PC_61_BM/ Al	60	8,180	7.75 × 10^11^ ^b)^	[56]
ITO/PEDOT:PSS/Al_2_O_3_/ P3HT:PC_71_BM/ Al	60	251,000	9.73 × 10^13^ ^a^ ^)^	[57]
ITO/PNDIT–F3N/ P3HT:PC_71_BM/ Al	−10	1,680	3.30 × 10^13^ ^b)^	[58]
ITO/PEDOT:PSS/ P3HT:PC_71_BM/ Al	−19	121,000	2.00 × 10^13^ ^a^ ^)^	[20]
ITO/PEDOT:PSS/ P3HT:PC_71_BM/ Al	−19	115,800	2.17 × 10^14^ ^b)^	[59]

^a)^ Specific detectivity values calculated with actual noise current values.

^b)^ Specific detectivity values overestimated with the shot‐noise‐limited assumption.

Bandwidth measurements were performed to analyze the dynamic behavior of the optimized CsPbBr_3_‐NC‐free and CsPbBr_3_‐NC‐embedded PM‐OPDs for external light signals at various frequencies. Previous studies on PM‐OPDs show that the response speed of PM‐OPDs has a tradeoff relationship with their photoconductive gain (or EQE).^[^ ^6^, ^7^, ^17^ ^]^ Therefore, CsPbBr_3_‐NC‐embedded PM‐OPDs are expected to exhibit a relatively longer carrier lifetime and smaller bandwidth compared to CsPbBr_3_‐NC‐free PM‐OPDs. As shown in Figure 5b and S9 (Supporting Information), the hole lifetimes / bandwidths were 319.7 µs / 879 dB and 415.6 µs / 775 dB for the optimized CsPbBr_3_‐NC‐free and CsPbBr_3_‐NC‐embedded PM‐OPDs, respectively, which was consistent with our expectation. Assuming that there is no change in Δ*p*/Δ*n* and *µ*_h_/*µ*_n_, increase of lifetime (≈30%) will improve the gain by ≈30% according to Equation (1) in the manuscript, which is smaller than the actual enhancement. This implies that the proposed strategy also affect Δ*p*/Δ*n* or *µ*_h_/*µ*_n_, which will be handled later.

The *G–B* product is another figure‐of‐merit for PM‐OPDs, which is theoretically defined as

(7) $$G\cdot B\;=\frac{\tau_{r}}{\tau_{\mathrm{transit}}}\;\left(1+\frac{\Delta p}{\Delta n}\frac{\mu_{p}}{\mu_{n}}\right)\cdot \;\frac{0.35p}{\tau_{r}p_{t}}=\frac{0.35p}{\tau_{\mathrm{transit}}p_{t}}\;\left(1+\frac{\Delta p}{\Delta n}\frac{\mu_{p}}{\mu_{n}}\right)$$

where *p* and *p*_t_ are the free and trapped hole densities.^[^ ^6^ ^]^ Because the fabricated PM‐OPDs employ the same donor/acceptor materials and the same photoactive layer thickness, as confirmed by cross sectional images (Figure S10, Supporting Information), and the film crystallinity did not differ after the incorporation of CsPbBr_3_ NCs, as confirmed by 2D‐GIXD (Figure 2 and Table 1), *p*, *τ*_transit_, *µ*_p_, and *µ*_n_ can be treated as constants. Through thermal admittance spectroscopy analyses, as shown in Figure S11 (Supporting Information), the trap densities of states for the CsPbBr_3_‐NC‐free and CsPbBr_3_‐NC‐embedded PM‐OPDs were comparable, implying that *p*_t_ also can be treated as a constant. Thus, the *G–B* product is influenced only by Δ*p*/Δ*n*. The *G–B* product of the CsPbBr_3_‐NC‐embedded PM‐OPD (2.14 × 10^7^ Hz) was larger than that of the CsPbBr_3_‐NC‐free PM‐OPD (1.76 × 10^7^ Hz). This indicates that additional electrostatic interaction within the photoactive layer increases the effective number of trapped electrons as well as *τ*_r_, which in turn leads to the synergetic improvement of photoconductive gain generation. Note that the *G–B* product of the CsPbBr_3_‐NC‐embedded PM‐OPD is the highest among those of previously reported PM‐OPDs for which both the gain and bandwidth were measured under the same illumination conditions (e.g., the same wavelength and light intensity; in this case, 520 nm and 1.24 × 10^−5^ W cm^−2^, respectively).^[^ ^6^, ^7^, ^17^ ^]^

Furthermore, the LDRs, that is, the ranges in which the photoresponse characteristics of the device is maintained, were evaluated for the optimized CsPbBr_3_‐NC‐free and CsPbBr_3_‐NC‐embedded PM‐OPDs. With the NEP density as the lowest measurable light power density, the LDR can be expressed as follows:

(8) $$\mathrm{LDR}\;=\;20\log\left(\frac{P_{\max}}{P_{\mathrm{NEP}}}\right)$$

where *P*_max_ is the maximum value of the detectable power density and *P*_NEP_ is the power density extracted from the NEP.^[^ ^60^, ^61^ ^]^ Using the *P*_NEP_ values (1.45 × 10^−15^ and 8.40 × 10^−16^ W cm^−2^ for the CsPbBr_3_‐NC‐free and CsPbBr_3_‐NC‐embedded PM‐OPDs, respectively) calculated from the current spectral densities of the fabricated PM‐OPDs (Figure S6, Supporting Information) and the *P*_max_ values (1.24 × 10^−4^ and 1.24 × 10^−5^ W cm^−2^ for the CsPbBr_3_‐NC‐free and CsPbBr_3_‐NC‐embedded PM‐OPDs, respectively) extracted experimentally from the *J*_ph_ *–P* plots (Figure 4c,d), the LDR values were 219 and 213 dB for the CsPbBr_3_‐NC‐free and CsPbBr_3_‐NC‐embedded PM‐OPDs, respectively. A relatively lower LDR value was obtained for the CsPbBr_3_‐NC‐embedded PM‐OPD because the photoresponse characteristics of the CsPbBr_3_‐NC‐free and CsPbBr_3_‐NC‐embedded PM‐OPDs could not be measured under illuminations with power densities over 1.24 × 10^−4^ and 1.24 × 10^−5^ W cm^−2^, respectively, owing to their significantly high responsivities.

## 3 Conclusion

Trap engineering is a key factor for enhancing the performances of PM‐OPDs. Therefore, several approaches have been reported for embedding inorganic NCs or quantum dots into PM‐OPDs so that trapped charges can be trapped in deeper energetic quantum well structures.^[^ ^62^, ^63^, ^64^ ^]^ However, in these existing hybrid approaches, the role of the inorganic NCs or quantum dots was of an n‐type semiconductor; thus, the resulting performances were only comparable to those of PM‐OPDs composed of pure organic p‐ and n‐type semiconductors. In contrast to these previous studies, here we introduced ionic‐polarizable CsPbBr_3_ perovskite NCs as inorganic additives in the photoactive layer. 2D‐GIXD analyses and EQE evaluations were conducted to determine the optimal concentration of CsPbBr_3_ NCs in the blend precursor to maintain the morphology and crystallinity of deposited films. Then, drift–diffusion simulations combined with temperature‐dependent *J–V* characteristic analyses were performed to show that ionic‐polarizable CsPbBr_3_ perovskite NCs under a high reverse bias are responsible for the strong electrostatic interactions between polarized cations (mostly Cs^+^ and some Pb^2+^) and trapped electrons in the organic domains. Contrary to the interfacial electrostatic interactions induced by the electric double layer, which enabled only the interfacially trapped electrons to generate Coulombic attraction,^[^ ^6^, ^7^ ^]^ extra electrostatic interactions within the photoactive layer initiated by ionic‐polarizable CsPbBr_3_ NCs enabled all the trapped electrons in the entire depletion region to generate Coulombic attraction, significantly enhancing hole injection from the cathode. The fabricated hybrid PM‐OPDs exhibited exceptionally high performances with an EQE of 2840000%, specific detectability of 3.97 × 10^15^ Jones, and *G–B* product of 2.14 × 10^7^ Hz. In addition, they exhibited a low NEP of 7.56 × 10^−17^ W cm^−2^ and LDR of 219 dB, demonstrating the advantageous features of CsPbBr_3_ perovskite NC additives for PM‐OPDs.

## 4 Experimental Section

### Materials

ITO‐patterned glass substrates (10 Ω sq^−1^) were purchased from Omniscience. PC_71_BM and PFN–Br were purchased from 1‐Material (Canada). P3HT was purchased from Rieke Metals (USA). Cesium carbonate (Cs_2_CO_3_; 99.9%), oleic acid (OA; 90%), 1‐octadecene (ODE; 90%), oleylamine (OAm; 90%), lead bromide (PbBr_2_; 99.999% trace metals basis), hexane (anhydrous, 95%), methyl acetate (MeOAc; anhydrous, 99.5%), methanol, isopropanol, 1,2‐dichlorobenzene (DCB), Mucasol, and acetone were purchased from Sigma‐Aldrich (USA). All the purchased chemicals were used as received without further purification or treatment.

### CsPbBr_3_ Perovskite Nanocrystal Synthesis

In a three‐neck flask, 0.2035 g Cs_2_CO_3_ was mixed with 0.625 mL OA and 10 mL ODE and heated to 120 °C under vacuum conditions. As Cs_2_CO_3_ dissolved and reacted with OA, gas evolved continuously. During this process, Cs_2_CO_3_ and OA reacted to form cesium oleate. After the reaction was complete, the solution was stored under nitrogen gas. In another three‐neck flask, 0.69 mg PbBr_2_ was mixed with 5 mL ODE and heated to 120 °C under vacuum conditions. After 1 h, 0.5 mL OA, and 0.5 mL OAm were injected into the three‐neck flask. The two ligands were dissolved and dispersed PbBr_2_. After dissolution was confirmed, the temperature of the solution was increased to 170 °C under a nitrogen atmosphere. Subsequently, the synthesized cesium oleate solution (0.4 mL, 120 °C) was quickly injected into the PbBr_2_ solution at 170 °C. After 5 s, the heater was removed, and the solution was cooled to room temperature using an ice bath. The crude solution was mixed with 10 mL MeOAc and then centrifugated at 8500 rpm at 5 °C for 5 min. After removing the supernatant, 1.5 mL hexane was added to disperse the perovskite NCs. Further, 3 mL MeOAc was added to the solution, and the solution was centrifugated at 8500 rpm at 5 °C for 5 min to remove excess ligands. The supernatant was removed; the pellets were dispersed in hexane and centrifugated at 8500 rpm at 5 °C for 5 min. At this time, the CsPbBr_3_ perovskite NCs remained dispersed in the supernatant. The optical properties (absorption and photoluminescence spectra) and transmission electron microscopy images of the diluted CsPbBr_3_ NCs are shown in Figure S12 (Supporting Information).

### Thin Film Deposition and Characterization

The samples used for the 2D‐GIXD measurements were fabricated using Si++/SiO_2_ substrates. Before deposition of the PFN–Br interlayer, O_2_ plasma treatment was performed on the substrates to improve the surface hydrophilicity and enhance the wettability of the PFN–Br solution. The PFN–Br solution was prepared by dissolving PFN–Br in methanol:isopropanol (2:1, v/v) with a concentration of 0.4 mg mL^−1^ at room temperature. The PFN–Br solution was spin‐coated onto the O_2_‐plasma‐treated substrates at 5000 rpm, and substrates were subsequently annealed at 140 °C for 30 min to evaporate the residual solvents. P3HT:PC_71_BM:CsPbBr_3_ NC (100:1:X, w/w; X = 0, 2, 4, 6, 8, or 10) blend solutions were prepared by dissolving the appropriate ratios of the constituents in DCB. The blend solutions were spin‐coated on the PFN–Br‐coated substrates at 1000 rpm, and the films were thermally annealed at 150 °C for 10 min in a N_2_‐filled glove box. The 2D‐GIXD measurements were conducted using the PLS‐II 3C beamline at the Pohang Accelerator Laboratory (PAL) in the Republic of Korea. To observe the possible changes in film morphologies after the incorporation of CsPbBr_3_ NCs, atomic force microscopy (AFM) studies were performed for the CsPbBr_3_‐NC‐free (100:1:0, w/w) and CsPbBr_3_‐NC‐embedded (100:1:6, w/w) P3HT:PC_71_BM blend films, as displayed in Figure S13 (Supporting Information).

### Photodiode Fabrication

The ITO‐patterned glass substrates were cleaned via sequential sonication in Mucasol aqueous solution, distilled water, acetone, and isopropanol for more than 20 min each. The PFN–Br interlayer and photoactive layer were deposited, as described in the previous section, except for the total concentration. A total of 40 mg mL^−1^ was used for the actual photoactive layer deposition. The thicknesses of the photoactive layers were ≈185 nm. MoO_3_ (30 nm)/Ag (100 nm) electrodes were deposited onto the photoactive layers via thermal evaporation under a high vacuum (≈5 × 10^−6^ torr). The photoactive areas of the fabricated photodiodes were 0.09 cm^2^. Cross sectional transmission electron microscope images of the CsPbBr_3_‐NC‐free (100:1:0, w/w) and CsPbBr_3_‐NC‐embedded (100:1:6, w/w) PM‐OPDs are shown in Figure S10 (Supporting Information).

### Photodiode Characterization

The *J–V* characteristics and EQE spectra were measured using a combination of a SourceMeter (2450, Keithley, USA), monochromator (Oriel Cornerstone 130 1/8 m), and Xe arc lamp (300 W), controlled with home‐made LabView programs (National Instruments Corp., USA). A spectrum analyzer (35670A, Agilent, USA) and a current pre‐amplifier (SR570, Stanford Research, USA) were used with a modulated 520‐nm laser source to measure the current spectral densities. The LDR was measured using a SourceMeter combined with a 520‐nm laser source and various neutral density filters. The bandwidth and transient photoresponse spectrum were measured using a mixed signal oscilloscope (MSO44, Tektronix, USA), with a 520‐nm laser source. Time‐resolved current density analyses were conducted with the CsPbBr_3_‐NC‐based hole‐only device (ITO/PEDOT:PSS/CsPbBr_3_ NC/MoO_3_/Ag) to observe the current density relaxation and extract interface charge densities measured under various applied voltages (Figure S4, Supporting Information). All measurements were conducted in a N_2_‐filled glove box.

## Conflict of Interest

The authors declare no conflict of interest.

## Supporting information

Supporting Information

## Data Availability

The data that support the findings of this study are available from the corresponding author upon reasonable request.
