# Supplementary material for: Boosting the Performance of Photomultiplication‐Type Organic Photodiodes by Embedding CsPbBr3 Perovskite Nanocrystals
Source: Adv Sci (Weinh). 2023 Dec 8;11(7):2305349. doi: 10.1002/advs.202305349 (PMC10870029; doi:10.1002/advs.202305349)
Supplement: Supplementary file 1 — Supporting Information [file ADVS-11-2305349-s001.pdf]

ADVANCED SCIENCE
Open Access

Supporting Information

for *Adv. Sci.*, DOI 10.1002/advs.202305349

Boosting the Performance of Photomultiplication-Type Organic Photodiodes by Embedding $CsPbBr_3$ Perovskite Nanocrystals

Mingyun Kang, Dong Hyeon Lee, Juhee Kim, Geon-Hee Nam, Seyeon Baek, Seongmin Heo, Yong-Young Noh and Dae Sung Chung**

Supporting Information

Boosting the Performance of Photomultiplication-Type Organic Photodiodes by Embedding $CsPbBr_3$ Perovskite Nanocrystals

Mingyun Kang, Dong Hyeon Lee, Juhee Kim, Geon-Hee Nam, Seyeon Baek, Seongmin Heo, Yong-Young Noh, Dae Sung Chung**

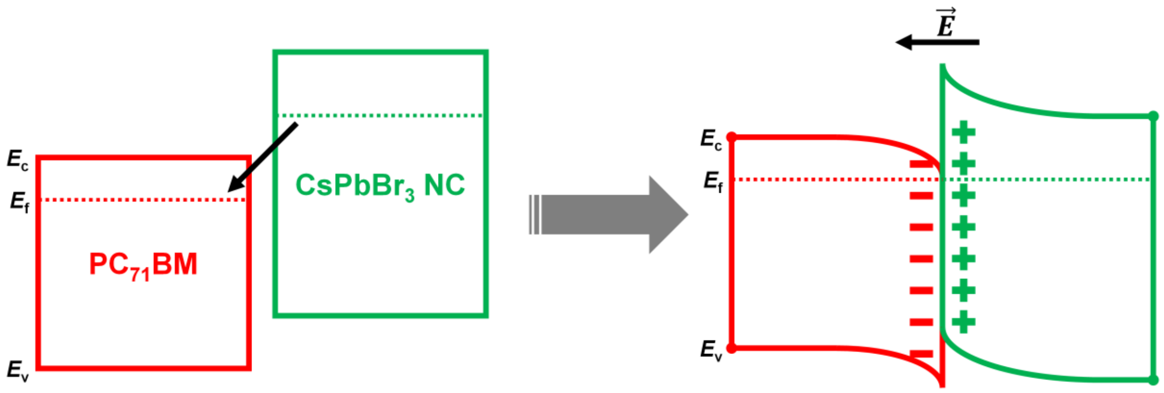

Figure S1. Energy band diagram for interfaces between $PC_{71}BM$ molecules and CsPbBr3 NCs after applying an external bias.

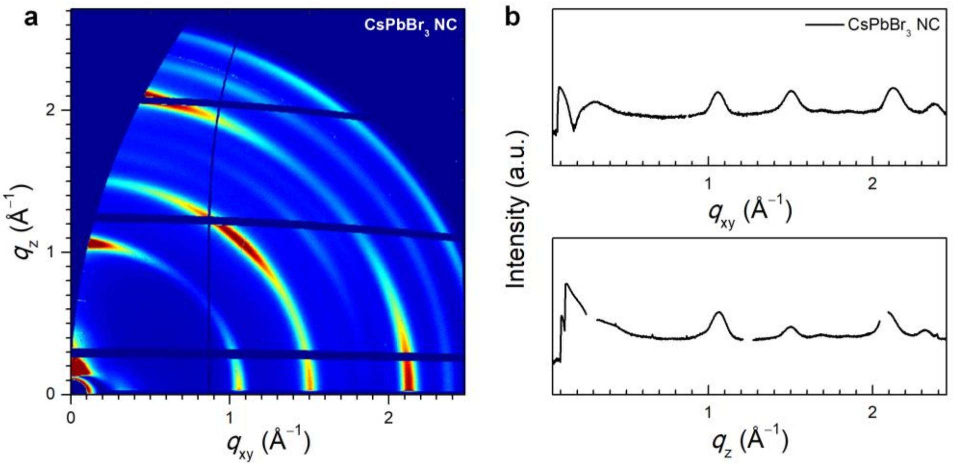

Figure S2. 2D-GIXD pattern (a) and line-cut profiles (b) of the $CsPbBr_3$ NC film.

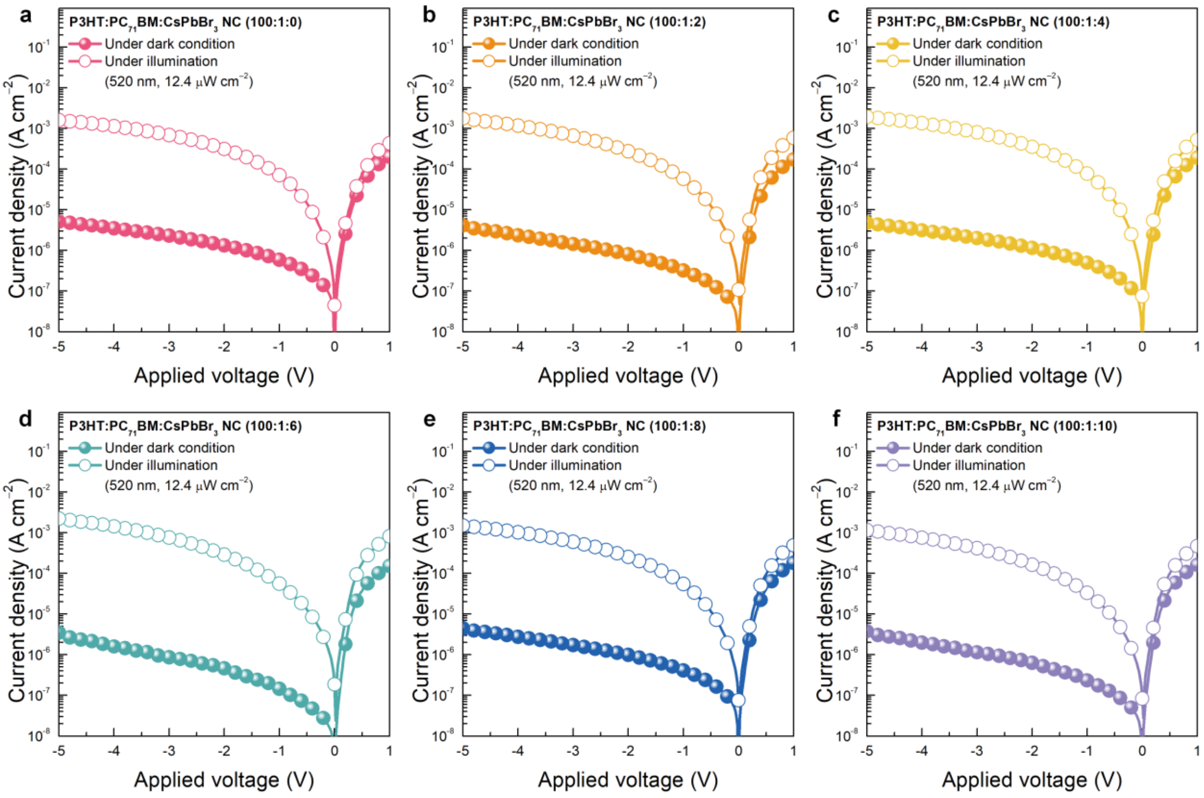

Figure S3. (a–f) *J*–*V* characteristics of the optimized PM-OPDs based on the P3HT:$PC_{71}BM$:$CsPbBr_3$ NC (100:1:X, w/w; X = 0, 2, 4, 6, 8, and 10) ternary blend films.

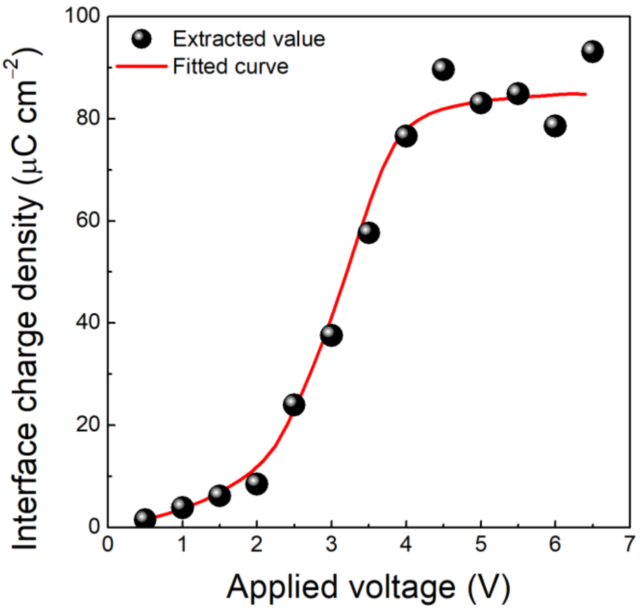

Figure S4. Interface charge densities measured under various applied voltages.

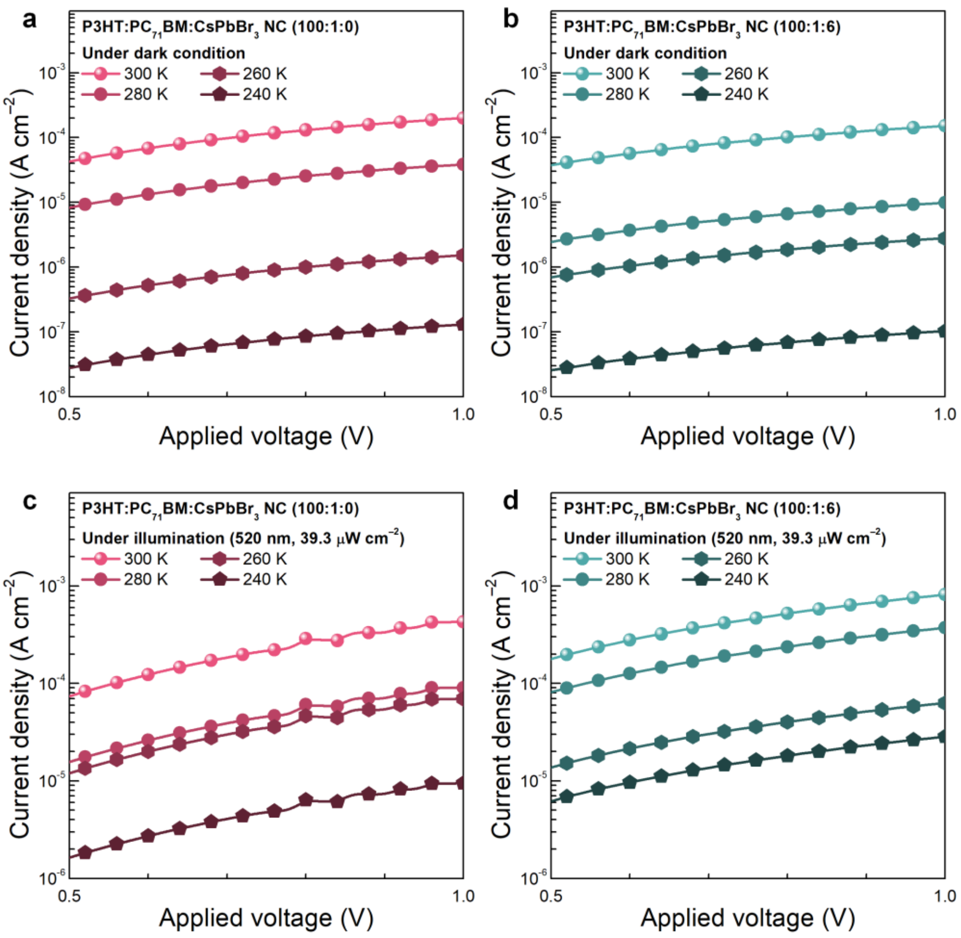

Figure S5. (a,b) Temperature-dependent dark *J*–*V* curves of the (a) $CsPbBr_3$-NC-free and (b) $CsPbBr_3$-NC-embedded PM-OPDs. (c,d) Temperature-dependent illuminated *J*–*V* curves of the (c) $CsPbBr_3$-NC-free and (d) $CsPbBr_3$-NC-embedded PM-OPDs.

Drift–Diffusion Simulations

We employed the drift–diffusion approximation method, described in detail in previous studies, for the numerical simulations in the Fluxim software (Setfos), in order to model the transport, trapping, injection, and generation of the charge carriers.[1–5] Single-level trap states were assumed for simplicity, similar to various previous theoretical analyses of the charge transport.[6,7] In addition, the typical boundary conditions described by Schottky were applied in the simulation.[7,8] Under these conditions, the electron and hole densities at the semiconductor/electrode interfaces are assumed to remain constant under external biases and illumination. Because the electrons were spatially localized owing to the small amount of $PC_{71}BM$ dispersed in the P3HT matrix, the hole and electron mobilities of P3HT reported in a previous study were considered, that is, 10^{-4} and 5×10^{-8} cm^2 V^{-1} s^{-1}, respectively.[1,3] Based on the assumption that the Schottky junction between the ITO/CPE and P3HT can be transformed to a pseudo-Ohmic junction under illumination owing to band bending, different work function values were used for the dark and illuminated conditions;[9–13] these conditions were determined through temperature-dependent analyses. Optical parameters such as the refractive indices and extinction coefficients of the constituting layers were obtained from literature or measurements.[15] Details of the simulation parameters are presented in Table S1.

Table S1. Summary of parameters used in numerical simulations.

**Temperature [K]**	300
**Thermionic work function of the cathode under dark condition [eV]**	$P3HT:PC_{71}BM:CsPbBr_3$ NC (100:1:0): 4.487<br>$P3HT:PC_{71}BM:CsPbBr_3$ NC (100:1:6): 4.491
**Thermionic work function of the cathode under illumination [eV]**	$P3HT:PC_{71}BM:CsPbBr_3$ NC (100:1:0): 4.852<br>$P3HT:PC_{71}BM:CsPbBr_3$ NC (100:1:6): 4.899
**Thermionic work function of the anode [eV]**	5.00
**HOMO level [eV]**	5.20

**LUMO level [eV]**	3.20
**N_0[a] [m^{-3}]**	10^{29}
**Dielectric constant**	2.7
**Charge carrier mobilities [$cm^2\ V^{-1}\ s^{-1}$]**<br>**(based on SCLC analyses)**	μ_e: 5×10^{-8}<br>μ_h: 1×10^{-4}
**Trap density[b] [cm^{-3}]**	10^{18}
**Trap energy depth [eV]**	P3HT:$PC_{71}BM$:$CsPbBr_3$ NC (100:1:0): 0.614<br>P3HT:$PC_{71}BM$:$CsPbBr_3$ NC (100:1:6): 0.699
**Electron/hole capture rate[b] [$cm^3\ s^{-1}$]**	10^{-13}
**Langevin recombination efficiency[c]**	1
**Non-radiative decay rate[c] [s^{-1}]**	10^5
**Generation efficiency[c]**	1
**Diffusion constant[c] [$cm^2\ s^{-1}$]**	0
**Annihilation rate[c] [$cm^3\ s^{-1}$]**	0
**Optical generation efficiency[c]**	0.66
**Pair separation[c] [nm]**	1.285
**Charge transfer type[c]**	Onsager–Braun
**Wavelength [nm]**	550 (single wavelength)
**Light intensity [W cm^{-2}]**	3.80×10^{-6}

[a]Work function values of the cathodes were determined by temperature dependent analyses

[b]Parameters used in Reference [1]

[c]Parameters used in References [3] and [15]

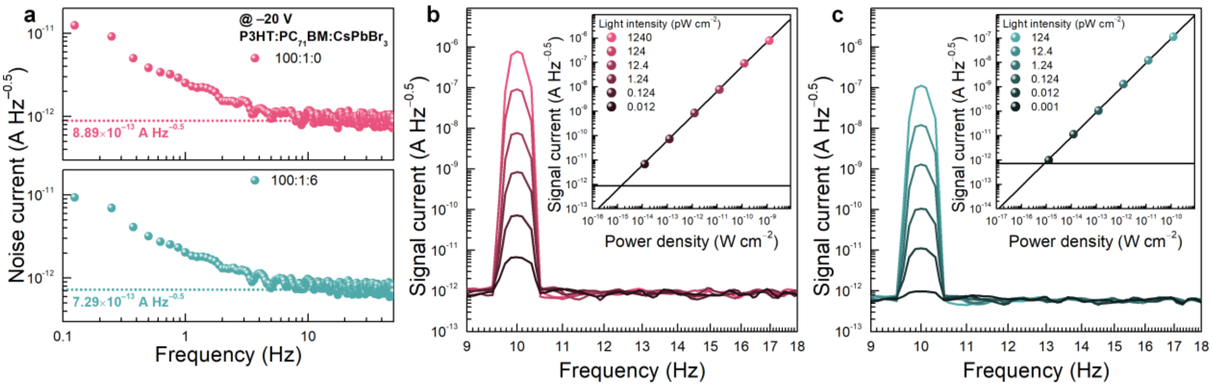

Figure S6. (a) Current spectral density plots of the optimized PM-OPDs based on the P3HT:PC$_{71}$BM:CsPbBr$_3$ NC (100:1:X, w/w; X = 0 and 6) ternary blend films, measured under a reverse bias of 20 V. (b,c) Current spectral density plots of the (b) CsPbBr$_3$-NC-free and (c) CsPbBr$_3$-NC-embedded PM-OPDs, measured under green light illumination (520 nm) with various light intensities and a modulation frequency of 10 Hz.

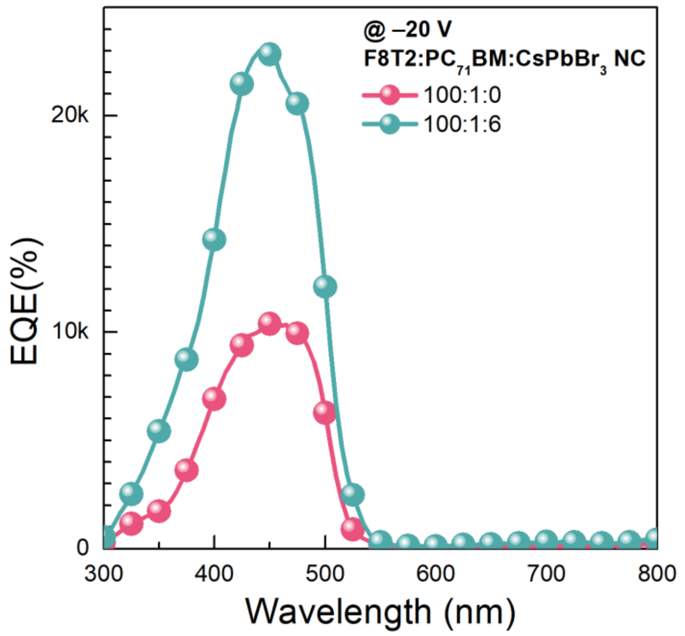

Figure S7. EQE spectra (at −20 V) of the optimized F8T2-based PM-OPDs with (100:1:6, w/w) and without (100:1:0, w/w) CsPbBr$_3$ NC.

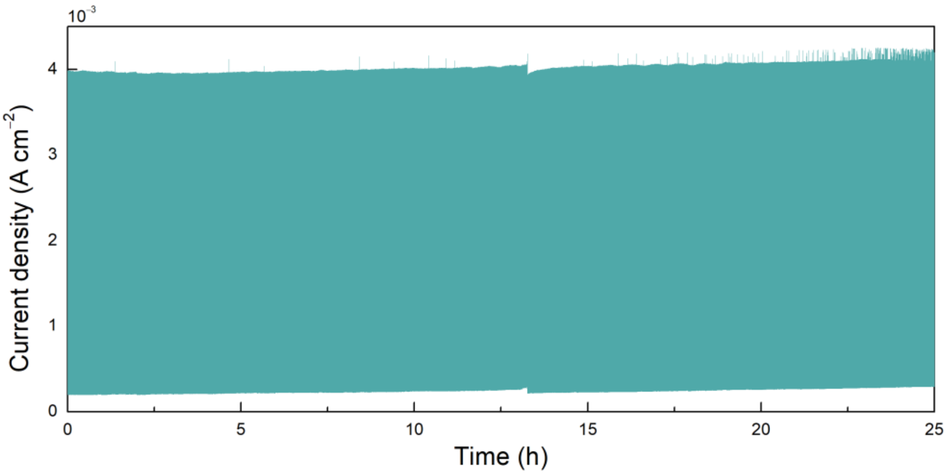

Figure S8. Operational stability of the $CsPbBr_3$-NC-embedded (100:1:6, w/w) PM-OPD. The measurement was conducted under the green (520 nm) illumination with a frequency of 1 Hz and intensity of 4.89×10^{-7} W cm^{-2}.

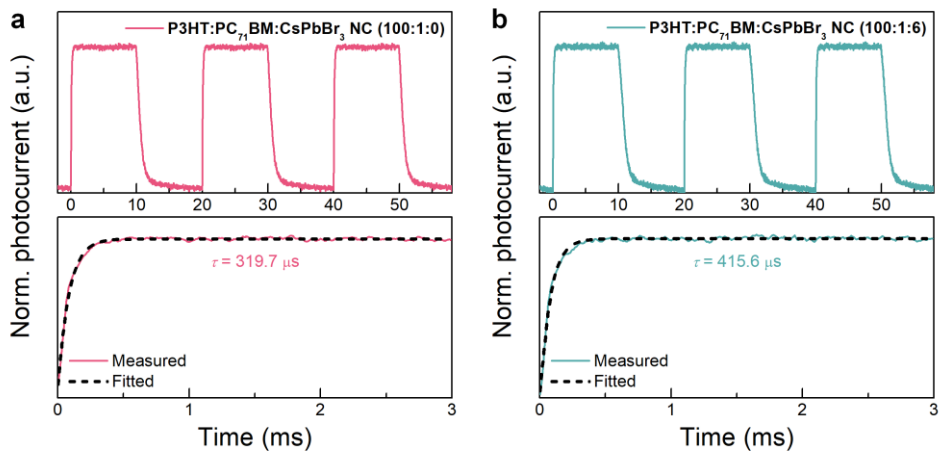

Figure S9. (a,b) Transient photoresponse spectra, measured at −20 V, and fitted curves of the (a) $CsPbBr_3$-NC-free (100:1:0, w/w) and (b) $CsPbBr_3$-NC-embedded (100:1:6, w/w) PM-OPDs. The measurements were performed under the green (520 nm) illumination with a frequency of 50 Hz.

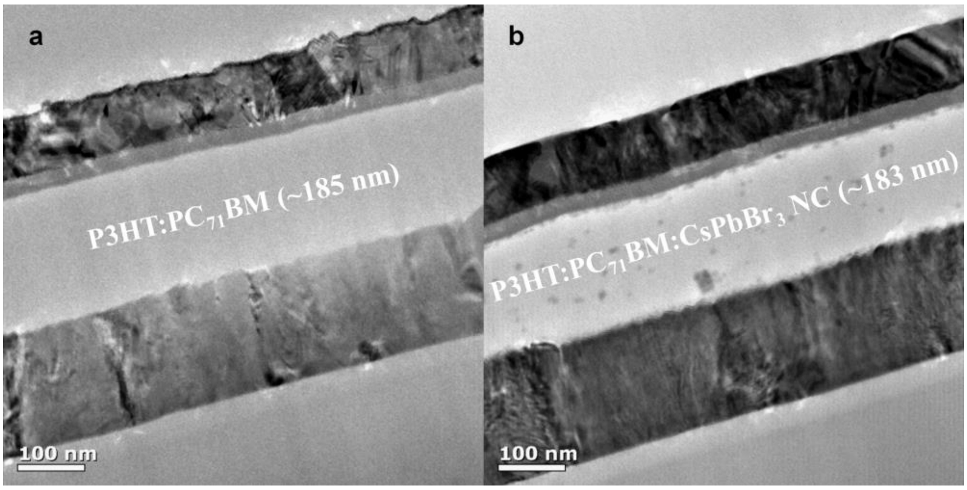

Figure S10. (a,b) Cross-sectional transmission electron microscope images of the (a) $CsPbBr_3$-NC-free (100:1:0, w/w) and (b) $CsPbBr_3$-NC-embedded (100:1:6, w/w) PM-OPDs. Because the degrees of electron transmission in PFN–Br and P3HT:$PC_{71}BM$ do not differ from each other, their interface is not distinguishable.

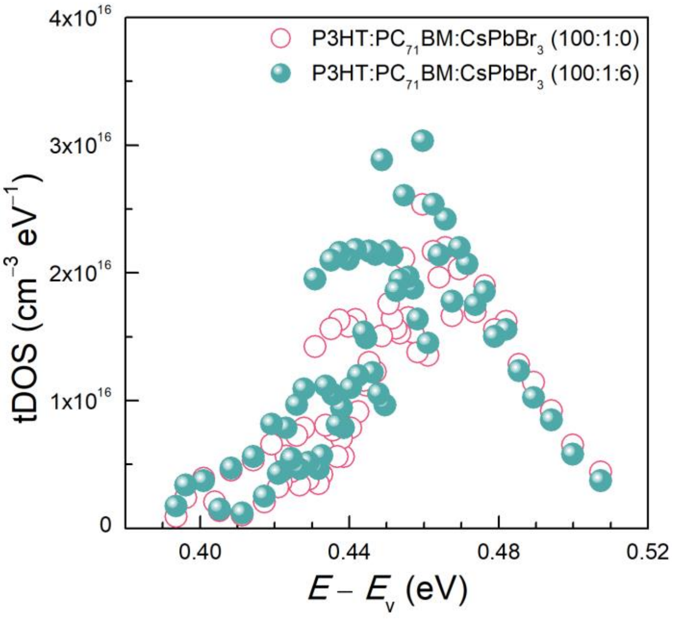

Figure S11. Trap state densities of P3HT:$PC_{71}BM$ (100:1, w/w) blend films with (100:1:6, w/w) and without (100:1:0, w/w) $CsPbBr_3$ NC, obtained using the thermal admittance spectroscopy method.

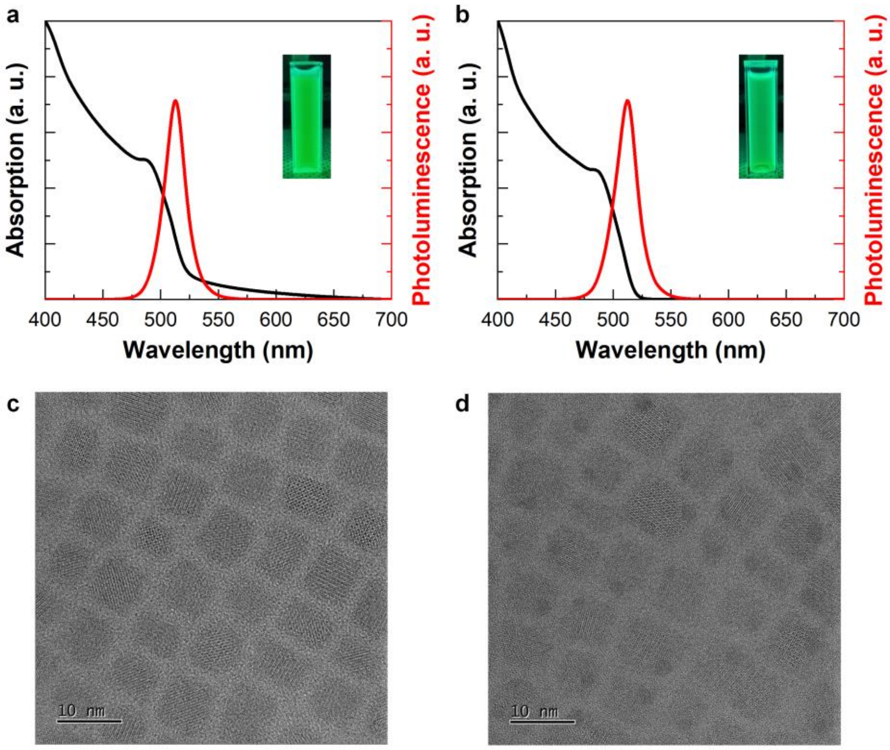

Figure S12. Optical properties and transmission absorption microscopy (TEM) images of $CsPbBr_3$ NCs. Absorption and photoluminescence spectra of (a) DCB-diluted and (b) hexane-diluted $CsPbBr_3$ NCs. TEM images of (c) DCB-diluted and (d) hexane-diluted $CsPbBr_3$ NCs.

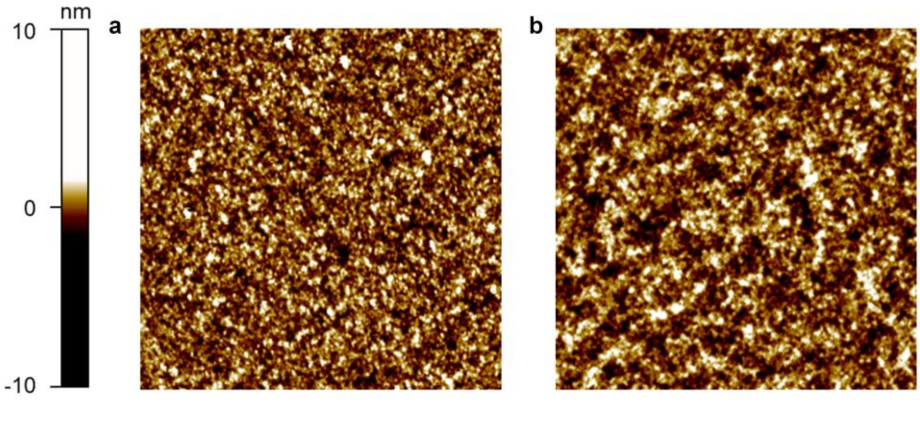

Figure S13. (a,b) Atomic force microscope images, with the scan size of (5×5) µm, of the (a) $CsPbBr_3$-NC-free (100:1:0, w/w) and (b) $CsPbBr_3$-NC-embedded (100:1:6, w/w) P3HT:PC_{71}BM blend films. The obtained roughness (R_{rms}) values were 0.759 and 0.824 nm for the $CsPbBr_3$-NC-free and $CsPbBr_3$-NC-embedded P3HT:PC_{71}BM blend films, respectively.

References

[1] M. Daanoune, R. Clerc, B. Flament, L. Hirsch, *J. Appl. Phys.* **2020**, *127*, 055502.

[2] S. Altazin, R. Clerc, R. Gwoziecki, G. Pananakakis, G. Ghibaudo, C. Serbutoviez, *Appl. Phys. Lett.* **2011**, *99*, 143301.

[3] R. Häusermann, E. Knapp, M. Moos, N. A. Reinke, T. Flatz, B. Ruhstaller, *J. Appl. Phys.* **2009**, *106*, 104507.

[4] L. J. A. Koster, E. C. P. Smits, V. D. Mihailetchi, P. W. M. Blom, *Phys. Rev. B* **2005**, *72*, 085205.

[5] Fluxim AG, Information about SETFOS: Semiconducting Emissive Thin Film Optics Simulator Software; http://www.fluxim.com/setfos-intro (accessed: February 2022).

[6] J. H. Jung, T. W. Kim, *J. Appl. Phys.* **2011**, *110*, 043721.

[7] K. Marcin, M. Daanoune, O. Françoic-Martin, B. Flament, O. Dhez, A. K. Pankey, S. Chambon, R. Clerc, L. Hirsch, *Adv. Electron. Mater.* **2018**, *4*, 1700526.

[8] D. A. Neamen, *Semiconductor physics and devices*, 3rd ed., McGraw-Hill, New York **2003**.

[9] M. Kang, S. Z. Hassan, S.-M. Ko, C. Choi, J. Kim, S. K. R. Parumala, Y.-H. Kim, Y. H. Jang, J. Yoon, D.-W. Jee, D. S. Chung, *Adv. Mater.* **2022**, *34*, 2200526.

[10] M. Kang, A. K. Harit, H. Y. Woo, D. S. Chung, *J. Mater. Chem. C* **2022**, *10*, 15160–15167.

[11] J. Kim, M. Kang, S. Lee, C. So, D. S. Chung, *Adv. Mater.* **2021**, *33*, 2104689.

[12] S. Yoon, G. S. Lee, K. M. Sim, M.-J. Kim, Y.-H. Kim, D. S. Chung, *Adv. Funct. Mater.* **2021**, *31*, 2006448.

[13] D. K. Neethipathi, H. S. Ryu, M. S. Jang, S. Yoon, K. M. Sim, H. Y. Woo, D. S. Chung, *ACS Appl. Mater. Interfaces* **2019**, *11*, 21211–21217.

[14] M. S. Jang, S. Yoon, K. M. Sim, J. Cho, D. S. Chung, *J. Phys. Chem. Lett.* **2018**, *9*, 8–12.

[15] V. D. Mihailetchi, H. Xie, B. de Boer, L. J. A. Koster, P. W. M. Blom, *Adv. Funct. Mater.* **2006**, *16*, 699–708.
